# Zinc, Magnesium, Selenium and Depression: A Review of the Evidence, Potential Mechanisms and Implications

**DOI:** 10.3390/nu10050584

**Published:** 2018-05-09

**Authors:** Jessica Wang, Phoebe Um, Barbra A. Dickerman, Jianghong Liu

**Affiliations:** 1 University of Pennsylvania School of Nursing, Philadelphia, PA 19104, USA; jeswang@sas.upenn.edu (J.W.); phoebeum@sas.upenn.edu (P.U.); 2 Harvard T. H. Chan School of Public Health, Boston, MA 02115, USA; bad788@mail.harvard.edu

**Keywords:** nutrition, micronutrient, diet, depression, zinc, magnesium, selenium, microbiota

## Abstract

Micronutrient deficiency and depression are major global health problems. Here, we first review recent empirical evidence of the association between several micronutrients—zinc, magnesium, selenium—and depression. We then present potential mechanisms of action and discuss the clinical implications for each micronutrient. Collectively, empirical evidence most strongly supports a positive association between zinc deficiency and the risk of depression and an inverse association between zinc supplementation and depressive symptoms. Less evidence is available regarding the relationship between magnesium and selenium deficiency and depression, and studies have been inconclusive. Potential mechanisms of action involve the HPA axis, glutamate homeostasis and inflammatory pathways. Findings support the importance of adequate consumption of micronutrients in the promotion of mental health, and the most common dietary sources for zinc and other micronutrients are provided. Future research is needed to prospectively investigate the association between micronutrient levels and depression as well as the safety and efficacy of micronutrient supplementation as an adjunct treatment for depression.

## 1. Introduction

Micronutrient deficiencies and depression are major global health problems, with more than two billion people in the world estimated to be deficient in key vitamins and minerals [1] and more than 300 million people suffering from depression [2]. Micronutrients have been consistently linked with health outcomes such as cognitive functioning [3,4], cancer [5,6], obesity [7,8], and immune functioning [9,10]. However, the role of micronutrients in the etiology and progression of depression remains unclear. Given that micronutrient deficiency is both prevalent and modifiable, even a modest association with risk of depression would be of public health interest.

Micronutrient deficiencies may play a role in the development of depression, and several studies have explored micronutrient supplementation as an adjunct to antidepressant therapy. Zinc and magnesium have been most commonly studied with respect to depression, and it has been suggested that these micronutrients might influence depression through similar biological mechanisms. Recent studies have suggested that selenium may also play a role in the development of depression, although evidence is sparse and inconsistent. 

The aim of the present review is to (1) examine empirical evidence of the association between micronutrients (zinc, magnesium, selenium) and depression; (2) discuss possible mechanisms of action; and (3) explore the clinical implications of such findings. As micronutrient deficiency and mental health are of great global public health importance, understanding the possible roles of micronutrients in depression will help elucidate the mechanisms underlying this condition and inform primary and secondary prevention strategies. 

## 2. Zinc

Zinc is an essential trace element important for many biochemical and physiological processes related to brain growth and function [11,12], as well as cellular metabolism [13,14]. Zinc is acquired through dietary intake of foods such as red meat, oysters and crab, and zinc deficiency can occur with reduced intake, insufficient absorption, and/or increased zinc utilization or expenditure. Normal serum zinc levels range from 0.66 to 1.10 μg/mL in adults [15]. The balance of intracellular and extracellular zinc levels is crucial for maintaining zinc homeostasis in many brain regions, including those involved in the physiopathology of depression, such as the hippocampus, amygdala, and the cerebral cortex [13,14,16]. 

An association between zinc and depression was first suggested in the late 1980s [17]. Since then, the association between zinc and depression has been extensively studied in both animals and humans. Rodent studies have reported associations between zinc deficiency and depressive symptoms [18,19,20,21]. Researchers have also reported lower serum zinc levels in animals more resistant to antidepressant treatment [18,20]. Observational studies have supported these findings [14,22,23,24]. A meta-analysis of 17 observational studies found that blood zinc concentrations were approximately 0.12 µg/mL lower in depressed subjects than in control subjects [25]. Cross-sectional studies among female adolescents [22,23], postmenopausal women [26] and patients on hemodialysis [27] have reported a positive association between zinc deficiency and depression severity. Interestingly, in their 2012 cross-sectional study, Maserejian et al. [14] found an association between zinc deficiency and depressive symptoms among women, but not men. A prospective cohort study similarly found no significant association between dietary zinc intake and the risk of depression among middle-aged men [28]. 

Intervention studies in both humans and rodents involving dietary or supplemental zinc have reported antidepressant-like and mood-enhancing activities of zinc [29,30,31]. In animal models, adult rats fed a zinc-deficient diet demonstrated more depressive symptoms than adult rats fed a zinc-sufficient diet, as assessed by the forced swim test, the tail suspension test as well as demonstrated anorexia and anhedonia [20,21]. Similarly, depressive symptoms induced in mice through chronic restraint stress (CRS) were alleviated by treatment by zinc (30 mg/kg) or imipramine, a traditional antidepressant [32]. Randomized controlled trials among individuals with depression have demonstrated decreases in depressive symptoms when supplementing antidepressant drug treatments with zinc compared to antidepressants alone [29,31]. Among healthy young women, those who received zinc and multivitamin supplements showed greater reductions in depression-dejection scores of the Profile of Moods State (POMS) assessment than those who had only received multivitamin supplementation [30]. 

### 2.1. Mechanisms

The potential mechanisms underlying the association between low serum zinc and depression remain unclear, but may involve the regulation of neurotransmitter, endocrine and neurogenesis pathways. Such mechanisms are outlined in Table 1.

In the hippocampus and cortex, zinc ions regulate synaptic transmission or act as neurotransmitters [33], modulating many ligand- and voltage-gated ion channels [34,35,36,37,38,39,40]. Disruption of zinc homeostasis in these regions has been implicated in many disturbances in cognition, behavioral and emotional regulation [41] through mechanisms of decreased neurogenesis [42,43] and neuronal plasticity [43].

Zinc deficiency has also been implicated in the endocrine pathway of depression. Takeda et al. [44] reported that a zinc-deficient diet induced high levels of serum cortisol concentration in rats. Persistently high levels of cortisol have been implicated in the development of depression via hyperactivity of the hypothalamic–pituitary–adrenal (HPA) axis [45,46]. Increased plasma cortisol levels could, therefore, potentially mediate the relationship between zinc deficiency and depression.

Further, recent research has highlighted the role of zinc transporters (ZincTs) and zinc-sensing GPR39 receptors in the development and treatment of depression [47]. Zinc transporter-3 knockout mice lack vesicular zinc and demonstrated fewer proliferating progenitor cells and immature neurons [48]. A reduced hippocampal volume has been extensively reported in association with depression [49,50], thus implicating the disruption of Zinct-3-dependent neurogenesis in the etiology of depression. In addition, GPR39 receptors have been increasingly associated with the serotonergic system, as recent studies have established links between GPR39 proteins and serotonin synthesis [51] and receptor signaling [52]. Moreover, GPR39 receptors have also been reported to play a role in the action of antidepressants. GPR39 knockout mice have been shown to be resistant to the normalizing effects of imipramine and escitalopram in the forced swim test (FST) [53]. Moreover, studies have shown that the binding of zinc to GPR39 receptors activates downstream cyclic AMP-response element (CRE)-dependent gene transcription, resulting in higher levels of brain-derived neurotrophic factor (BDNF) in the hippocampus and cortex [54]. The action of zinc mimics the actions of traditional antidepressants, and previous studies have shown normalization of low BDNF levels in depressed patients treated with antidepressants [55,56].

Another possible reason for the antidepressant effects of zinc may be the anti-inflammatory and antioxidant properties of zinc supplementation. Previous studies have reported that zinc supplementation decreases C-reactive protein (CRP) levels in humans [57,58]. Increased CRP levels have been previously associated with depression [59,60], and a recent study found that the effectiveness of the antidepressant, agomelatine, was associated with a reduction in CRP levels [61]. Similarly, zinc has demonstrated protective effects against lipid peroxidation [62,63]. Recent evidence has supported a relationship between lipid peroxidation and major depression [64], suggesting that the observed antidepressant properties of zinc result, in part, from its antioxidant effects.

Lastly, the potential antidepressant properties of zinc may be related to its function as an antagonist of the glutamatergic *N*-methyl-d-aspartate (NMDA) receptor and involvement in the l-arginine–nitric oxide (NO) pathway as a nitric oxide synthase (NOS) inhibitor. NMDA has been therapeutically targeted in clinical and preclinical studies of depression treatment, as growing evidence supports the presence of disrupted glutamate homeostasis and neurotransmission in depressed subjects [65]. In a study that measured depressive symptomology in mice, both blockage of NMDA receptors and addition of NOS substrate independently negated the beneficial effects of zinc–chloride on the reduction of depressive symptoms, suggesting that the antidepressant properties of zinc–chloride may have been partially mediated by zinc’s inhibition of NOS and NMDA receptors [66]. 

### 2.2. Discussion and Implications

Several methodological considerations underlie the evaluation of the research studies reviewed here. First, the measurement error of zinc status should be considered. While the measurement of serum zinc levels has been shown to be a useful biomarker of population zinc status, its reliability as an indicator of individual zinc status has not been demonstrated [67]. The relationship between serum zinc levels and depression could be partially explained by reverse causation, whereby depression influences the intake [68], bioavailability or biological regulation of zinc [20,32,69]. Oxidative stress and its accompanying immune-inflammatory response have been linked to the pathophysiology of depression [70]. In response to oxidative stress, levels of pro-inflammatory cytokines (e.g., interleukin 1 (IL-1) and IL-6) increase and, in turn, decrease of the level of albumin and increase the synthesis of metallothioneins [71]. Albumin is the main zinc transporter [72], and a decrease in albumin coupled with an increase in metallothioneins may compound to decrease serum levels of zinc. Future studies should include oxidative stress markers to further assess the directionality of the relationship between zinc status and depression status.

Finally, potential confounding by socioeconomic status and diet should be considered, as these factors could influence both zinc status and the risk of depression. Hair cortisol and parental education status have been found to be associated with hair zinc levels in a population of Canadian preschoolers [73], thus pointing towards a possible mechanism through which socioeconomic status could influence both zinc levels and depression. Dietary factors, such as the consumption of phytates, a compound present in many grains, have been shown to reduce zinc absorption in the intestine [74]. Furthermore, as zinc is primarily consumed through red meats and seafood, diets that limit the consumption of these foods (e.g., vegetarianism, veganism) may alter serum zinc levels. 

Further prospective studies are needed to investigate potential biologic mechanisms that may underlie the association between zinc and depression. Zinc deficiency may increase the vulnerability to psychological stress by depressing levels of neurogenesis and plasticity, and maintaining electrophysiological balance in various brain regions. These psychological and biological changes may act in concert to influence the development of depression, which itself could further reduce serum zinc levels. If evidence for a causal effect of zinc on depression risk accumulates, future studies exploring the safety and effectiveness of zinc as a potential supplement to antidepressants could also be warranted. Future intervention treatments should note that the presence of excess zinc can be potentially problematic. Secondary copper deficiency has been demonstrated as a potential consequence of a high dietary intake of zinc [75]. As a result, it is recommended that dietary zinc intake is limited to the recommended amount or that zinc supplementation is coupled with adequate copper supplementation. In the Age-Related Eye Disease Study, supplementation of zinc was given with a small amount of copper (80 mg zinc oxide, 2 mg cupric oxide) [76]. 

## 3. Magnesium

Magnesium is a micronutrient that is essential for the proper activity of many biochemical and physiological processes, including DNA replication, transcription and translation [77,78]. It is a bivalent intracellular cation that acts as a coenzyme or an activator for over 300 enzymatic systems, many of which are important for proper brain function [79]. Magnesium is usually consumed through nuts, seeds, green leafy vegetables and whole grains. Normal serum magnesium levels range from 0.62 to 1.02 mmol/L [80]. A 2005 study that leveraged dietary surveys suggested that 68% of Americans consume less than the recommended daily allowance of magnesium [81]. Magnesium levels are important for central nervous system (CNS) function and may play a role in Alzheimer’s disease, diabetes, stroke, hypertension, migraines and attention deficit hyperactivity deficit [82]. Previous studies have associated magnesium with various brain regions in the limbic system [83], thus implicating a possible role for magnesium in the etiology and progression of depression.

A positive association between magnesium deficiency and depression has been documented in both animal and human studies. Mice subjected to magnesium deficient diets have shown behavioral deficits associated with depression [84,85,86]. Likewise, cross-sectional studies [26,87,88,89] have reported an inverse relationship between depressive symptoms and magnesium levels and magnesium intake, which persisted after adjustment for age, body mass index, and education. However, prospective cohort studies have failed to find an association between magnesium status and later risk of depression. A study performed in the SUN Mediterranean cohort of 15,863 men and women without any history of depression found no significant association between magnesium intake, as assessed by diet, and risk of depression 10 years later [90]. Another study of approximately 13,000 Spanish university graduates free of depression at baseline reported an inverse association between magnesium intake and depression incidence 6 years later [91].

Some intervention studies have suggested a beneficial role of magnesium supplementation in the treatment of depression [92,93], while others have not [94,95]. A recent randomized clinical trial in a population of adults diagnosed with mild-to-moderate depression found that the consumption of 248 mg of magnesium per day for 6 weeks resulted in a clinically-significant 6 point decrease (*p* < 0.001) in depressive symptoms, as measured by the Patient Health Questionnaire-9 (PHQ-9) compared to those receiving a placebo treatment [93]. Similarly, a randomized controlled trial of 60 individuals with depression and hypomagnesemia demonstrated that daily consumption of 500 mg magnesium oxide led to significant improvements in Beck Depression Inventory scores, compared to individuals with depression and hypomagnesemia who received a placebo [92]. However, intervention studies among postpartum women [95] and an elderly population with hypomagnesemia [94] found no effect of magnesium supplements, of 328 mg/day and 50 mg/day, respectively, on the depression statuses of these individuals. 

### 3.1. Mechanisms

The biological mechanisms that potentially underlie the association between low serum magnesium levels and depression remain unclear but may involve the central nervous system, stress axis, and oxidative pathways. These mechanisms are outlined in Table 1.

Magnesium deficiency has been shown to lead to changes in the functioning of the central nervous system (CNS), especially in the glutamatergic transmission in the limbic system and cerebral cortex [54]—brain regions that play important roles in the etiopathogenesis of depression [96,97,98]. Magnesium is particularly well known for its importance as an antagonist of the NMDA glutamate receptor, which has long been understood as a key player in synaptic potentiation, learning and memory [99,100]. However, despite magnesium’s well-known involvement in the voltage gating function of the receptor, evidence pointing towards direct magnesium-induced changes in NMDA channels in the expression of depression-like behavior is scarce. A recent mouse study found that dietary magnesium restriction reduced levels of the GluN1 NMDA receptor subunit in the amygdala and hypothalamus [101], a phenomenon that mirrors the GluN1 reduction response to chronic stress [102]. Further, because NMDA channels mainly conduct calcium and sodium currents, a depletion of magnesium could allow for excess calcium current. Evidence supports the possibility that magnesium deficiency disrupts neuronal function by means of increasing neuronal calcium flow, thereby resulting in increased nitric oxide, a toxic reactive oxygen species that leads to neuronal swelling and death [103,104,105]. While the mechanism has yet to be elucidated, studies have demonstrated that the ameliorative effects of magnesium on depressive symptoms in mice can be reversed by NMDA-receptor agonists [106], thus pointing to a possible interaction between magnesium and the NMDA receptor as a therapeutic target for the treatment of depression.

Another possible mechanism for magnesium’s protective effect against depression could involve magnesium’s modification of the stress response. Magnesium’s ability to reduce the release of adrenocorticotrophic hormone (ACTH) and modulate adrenocorticotropic sensitivity to ACTH is preventative against the hyperactivation of the HPA axis. Dysregulation of the HPA axis in adults has been robustly linked to stress and depression; elevated cortisol and dysregulated HPA activity are highly over-represented in depressed populations [107,108]. Glucocorticoids have been continuously demonstrated to exhibit neurotoxic effects in the hippocampus, thus suggesting a role for excess glucocorticoids in the hippocampal cell death observed in depression [109,110]. If HPA axis dysfunction plays a mechanistic role in depression, magnesium deficiency could be a risk factor making individuals vulnerable to chronic elevated cortisol and its neurodegenerative effects.

Magnesium’s role in the gut microbiota (GM) has been of recent interest, as alterations in GM have been linked to depression [111,112]. Magnesium-induced changes in microbiota have also been associated with changes in the oxidative and inflammatory response, characterized by increased cytokines and biomarkers of cellular stress [113]. A recent study examining depression in mice demonstrated that 6 weeks of a magnesium-deficient diet induced depressive symptoms in the FST, which was associated with changes in GM and hippocampal interleukin-6 [86]. Previous studies have also demonstrated an inverse association between dietary magnesium intake and levels of inflammatory markers, such as serum C-reactive protein, interleukin-6 and tumor necrosis factor-α receptor 2 [114,115]. As evidence for the potential roles of inflammation and oxidative stress in the pathogenesis [116,117] and the progression of depression [118] continues to accumulate, it may be important to consider magnesium’s immune modulatory role.

Magnesium could potentially exert antidepressant effects through its role in serotonergic, noradrenergic and dopaminergic neurotransmission [119,120], increased expression of BDNF [121] and modulation of the sleep–wake cycle through augmentation of the biosynthesis of melatonin [122]. 

### 3.2. Discussion and Implications

Overall, the majority of evidence supports an inverse association between magnesium and the development of depression, as well as the antidepressant properties of magnesium. Methodologically, it is important to note that erythrocyte magnesium levels have been demonstrated to be more reliable in determining magnesium deficiency than serum levels [123]. Because only about 1% of total body magnesium is typically found extracellularly in the serum, serum magnesium levels are not necessarily representative of total body magnesium or the concentration of magnesium found intracellularly that is available for cellular use [124]. Magnesium homeostasis is maintained by the intestine, the bones and the kidneys; magnesium should still be consumed regularly in sufficient amount to prevent deficiency [125]. As a result of active biological regulation of magnesium levels, magnesium deficiency is likely an indicator of poor nutrition or ailments that affect magnesium absorption or excretion, such as diabetes mellitus [125]. The reviewed studies using serum magnesium can thus be viewed as reliable indicators of persistent hypomagnesemia in individuals, rather than a daily fluctuation in magnesium intake. 

Potential confounding by obesity, comorbidities, medication or diet should be considered, as these factors may be common causes of a low magnesium status and depression. Potential confounding by vitamin D and calcium levels is also possible. Magnesium is known to play a role in calcium balance as well as vitamin D metabolism [126], and dysregulation of these two compounds has also been implicated in depression [127,128]. Lastly, reverse causation may again be considered, whereby magnesium deficiency may be secondary to depression-related behavioral changes, such as reduced food intake [20]. 

Going forward, additional studies are needed to investigate the potential mechanisms that may underlie the association between magnesium and depression, as well as individual-level factors that might explain the variable associations found for magnesium supplementation. The evaluated studies varied with regard to magnesium dosage, ranging from 50 to 500 mg/day, and there is no clear trend between magnesium dosage and antidepressant effects. Similarly, the studies also varied with regard to patient population and duration of treatment as well as the unreliability of magnesium intake reports, which could, in turn, affect the discrepant findings. Furthermore, there is no observed relationship between the effect of magnesium supplementation and pre-treatment with magnesium or depressive status. Additional intervention studies might clarify whether magnesium supplementation can confer any benefits in the treatment of depression. 

## 4. Selenium

Selenium is an essential trace element that is vital for the proper functioning of several selenoproteins involved in antioxidant defenses within the brain and nervous system [129,130,131]. Currently, the recommended daily allowance of selenium is 55 μg/day [132]. Optimal serum selenium levels are defined as being between 70 μg/L and 90 μg/L [133]. As the source of selenium intake is through the consumption of grains, selenium intake is highly dependent on the selenium content in food, which is, in turn, dependent on the selenium content of the soils in which it is grown. As a result, selenium deficiency often results from suboptimal presence in regional soil, thus making selenium deficiency often an endemic problem. It is estimated that one in seven people have low dietary selenium intake [134], and selenium deficiency has been implicated in a variety of conditions, such as renal disease [135] and obesity [136].

Given its neuromodulatory role in brain function [137,138,139], recent studies have investigated a relationship between selenium levels and depression. A rodent study found an association between selenium deficiency and decreased BDNF concentrations [140]. As a neurotrophic factor that has been extensively associated with the pathophysiology of major depressive disorder [141,142], it is plausible that BDNF concentrations could mediate the relationship between selenium deficiency and depression. In an intervention study performed on mice, Brüning et al. [143] showed that the administration of m-trifluoromethyl-diphenyl diselenide (m-CF_3_–PhSe)_2_, a multi-target selenium-based compound, reduced depressive symptoms as measured by immobility time in a forced swimming test (FST), in female mice, suggesting a potential antidepressant effect of selenium.

Observational studies have also investigated the relationship between selenium and depressive symptomology or risk of depression but have provided inconsistent results. A cross sectional study performed in a middle-aged population in West Texas demonstrated an inverse relationship between selenium level and depressive symptoms as measured by the Geriatric Depression Scale (GDS) [144]. Similarly, data from a nested case-control study on 1494 women aged 20–89 years reported that lower dietary selenium intake (<8.9 μg/day) was associated with a higher risk of developing major depressive disorder [139]. However, the results of two cross-sectional studies performed among a geriatric population in rural China [137] as well as a population of hemodialysis patients [145] found no significant association between selenium levels and depression scores after controlling for chronic kidney disease and cognitive function. Conversely, several studies have found a positive association between selenium serum levels and depressive symptomology. In a cross-sectional study using toenail biomarkers from 3735 participants aged 20–32 years, Colangelo et al. [146] found that higher levels of selenium exposure as assessed through toenail clippings were associated with the presence of elevated depressive symptoms. The idea that an “optimal range” with respect to depressive outcomes may exist for selenium levels was supported by Conner et al.’s [147] cross sectional study that found increased depressive symptomology below and above the serum selenium levels of 82 and 85 μg/L, with depressive symptomatology at its lowest at 85 μg/L. 

Intervention studies of selenium supplementation in humans have reported similarly inconsistent findings. A randomized control trial among 166 Iranian women found that selenium supplementation during pregnancy was associated with increased selenium serum levels as well as lower scores on the Edinburgh Postnatal Depression Scale (EPDS) compared to those receiving placebo after 8 weeks of treatment [138]. However, Rayman et al. [148] reported the results of a randomized control trial to evaluate the effect of selenium supplementation on mood using the Profile of Moods States-Bipolar Form (POMS-BI) questionnaire and found that supplementation of selenium significantly increased plasma selenium levels without influencing mood scores after six months of supplementation. 

### 4.1. Mechanism of Action

The association between selenium and depression has been less explored than the associations between zinc and magnesium with depression. However, research has suggested several possible hypotheses regarding selenium’s mood-enhancing effects, including its role in maintaining metabolic, oxidative and central nervous system functioning. These are outlined in Table 1.

Selenium’s modulatory effects on metabolism may influence an individual’s susceptibility to developing depression. Selenium, which is incorporated into iodothyronine deiodinases (DIOs), is essential for the proper synthesis and metabolism of thyroid hormones. It has been long recognized by clinical investigators that thyroid function is associated with neuropsychiatric manifestation, such as mood disorders, cognitive dysfunction and other psychiatric symptoms [149]. Selenium deficiency and resulting deregulation of thyroid function may play a role in the development of depression [150].

The results from our review also indicate that an increasing amount of evidence points towards an “optimum range” of serum selenium levels in relation to depressive symptomology [146,147]. Studies have found that both high and low selenium levels have been linked with dysregulation of oxidative and inflammatory pathways, offering another potential mechanism that could explain the observed association between selenium levels and depression. Selenoproteins, such as glutathione peroxidases, thioredoxin reductases and selenoprotein P, are known to provide protection against lipoperoxidation and oxidative cell damage. A low selenium concentration has been associated with an increased level of pro-inflammatory cytokines, such as interleukin-6 (IL-6), C-reactive protein, and growth differentiation factor-5 (GDF-5) [151,152,153]. Additionally, a 2017 study demonstrated an association between low levels of cholesterol and increased risks of depression and suicidality [154], suggesting a possible role for selenium’s anti-lipoperoxidative actions in its protective effect against depression. However, at an optimum level of selenium supplementation, there is an enzymatic and protein saturation effect that sends excess selenium into a metabolic process to restrict the further creation of selenoproteins. The metabolites of this excess selenium metabolism have been demonstrated to be pro-oxidative and result in increased levels of damaging reactive oxygen species (ROS) [155] Recent studies have shown that depression is associated with increased levels of oxidative stress biomarkers, strengthening the hypothesis that oxidative stress and inflammation may be significant factors in the pathogenesis of depression [149]. In light of selenium’s dual antioxidant and pro-oxidative properties, it is conceivable that hyperactivity of oxidative and inflammatory pathways can contribute to the pathophysiology of depression.

Lastly, selenium could potentially exert antidepressant effects through its modulatory role in various neurotransmitter systems. Selenium has been found to have significant modulatory effects on the dopaminergic, serotonergic, and noradrenergic systems [156], which are all involved in the physiopathology of depression and other psychiatric illnesses [157]. Neurochemical data indicate that (m-CF_3_–PhSe)_2_ modulates the serotonergic system through mechanisms that involve selective inhibition of monoamine oxidase A (MAO-A), an enzyme implicated in 5-HT degradation, resulting in an overall increase of 5-HT availability in the synaptic cleft, contributing to its pharmacological effects [158]. Similarly, dopaminergic neurons vulnerable to oxidative stress have been shown to be modulated by selenoprotein, thus allowing selenium to play a preventative role in neurodegeneration [159]. While more studies are needed to clarify the relationship between selenium and depression, these findings suggest several plausible mechanisms through which selenium could be protective against depression.

### 4.2. Discussion and Implications

Studies examining the association between depression and selenium have been largely inconclusive. Overall, selenium deficiency seems to correlate with depression symptoms. However, this result was not observed in hemodialysis patients or in a rural elderly population in China. It is important to note that dialysis patients are at increased risk of selenium depletion due to low diffusion of selenium over the dialyzer membrane [160]. Moreover, comorbidities and other confounders, such as age and geographic location, may contribute to the discrepant findings. Alcohol is another potentially important confounder of this relationship as chronic alcoholism leads to lower plasma selenium through a reduction of selenium deposits as well as depression of selenoprotein expression and activity [161]. Longitudinal and cross sectional studies have further found associations between alcohol abuse and depression [162,163], In addition, zinc [25] and iron [164] may serve as confounders as both have been associated with depression status. Zinc has been shown to modulate the bioavailability of selenium, although the mechanism for this relationship is unclear [165]. 

Selenium exposure classification also varied within the reviewed studies. The majority of the reviewed studies utilized serum selenium or dietary intake of selenium as measurements of selenium levels. Selenium is regulated through excretion, and a direct relationship is evident between selenium intake and excretion [166]. As a result, selenium must be consumed regularly to maintain adequate selenium levels. However, the brain retains selenium better than any other tissue [167] and thus, measurement of selenium serum deficiency may not be indicative of brain selenium levels. Similarly, utilizing dietary recall incurs the risk of unreliability and recall bias that could potentially confound the results.

Further prospective investigations are needed to clarify the relationship between selenium and depression and to investigate reasons for the discrepant findings noted in this review. The inconsistent findings could be a result of different participant characteristics, such as geographic location, which plays a role in the amount of selenium available in local produce and subsequent selenium intake. Similarly, differing methods of the measurement of depression outcome as well as the duration of selenium treatment could potentially explain discrepant findings. For future intervention trials, it is important to note that high levels of selenium are toxic, as high dose selenium supplementation at or above 1600 μg/day has been shown to induce symptoms of selenium toxicity [168].

## 5. Conclusions

In this integrated review, we examined several lines of evidence, including animal, observational, and intervention studies, which provided evidence for a potential role of micronutrients in the development and progression of depression. The literature most strongly supports a role for zinc deficiency in increasing the risk of depression as well as the mood-enhancing effects of zinc supplementation in populations both with and without depression. While studies examining the magnesium–depression relationship have reported mixed findings, evidence generally supports a relationship between magnesium deficiency and the risk of depression as well as its antidepressant properties. The fewest number of studies have examined the link between selenium and depression, as most of the literature studying micronutrients has focused on zinc and magnesium. Studies examining the selenium–depression relationship have reported inconsistent findings, and future studies are needed to understand the effects of selenium deficiency on the etiology and treatment of depression. Although selenium deficiency is quite rare and regionally-specific, it has been recognized as an imminent public health problem due to the effects of climate change [169]. More prospective cohort and intervention studies are needed to assess the relationship between serum micronutrient levels and later risk of depression as well as the potential mechanisms underlying the observed associations. If evidence for a causal effect of these micronutrients on depression outcomes accumulates, the safety and efficacy of micronutrient supplementation as an adjust treatment for depression could also be explored. A balanced diet including adequate intake of foods containing zinc and other micronutrients could be an effective supplement to antidepressants for alleviating depressive symptoms.

## Figures and Tables

**Table 1:** Potential Mechanisms, Food Sources, and Normal Serum Levels of Zinc (Zn), Magnesium (Mg), and Selenium (Se).

	Potential Mechanisms		Food Sources	Normal Serum Levels in Adults
	Development of Depression	Antidepressant Action		
Zn	Increased cortisol; decreased neurogenesis and neural plasticity; disruption of glutamate homeostasis	*N*-methyl-d-aspartate (NMDA) antagonist; elevated expression of hippocampal and cortical brain-derived neurotrophic factor (BDNF)	Oysters, beans, nuts, red meat, certain types of seafood (crab and lobster), whole grains, fortified breakfast cereals, dairy products	0.66–1.10 μg/mL
Mg	Dysregulation of hypothalamic–pituitary–adrenal (HPA axis); increased Ca^2+^ in brain; increased inflammatory response	NMDA antagonist; serotonin, dopamine, noradrenaline modulation; increased BDNF expression; modulation of sleep–wake cycle	Green leafy vegetables (spinach), some legumes (beans and peas), nuts and seeds, whole grains	0.62–1.02 mmol/L
Se	Dysregulation of thyroid function; dysregulation of oxidative and inflammatory pathways	Serotonin, dopamine, noradrenaline modulation; attenuation of inflammation	Seafood, bread, grains, meat, poultry, fish, eggs	70–90 μg/L
